# Psychosocial aspects of continuous subcutaneous insulin infusion in children with type 1 diabetes in Egypt; a limited resources country perspective

**DOI:** 10.1186/s13098-022-00853-6

**Published:** 2022-06-11

**Authors:** Mona Hussein El Samahy, Nouran Yousef Salah, Mai Seifeldin Abdeen, Batrishia Rafat Kamel Falastin

**Affiliations:** 1 Department of Pediatrics, Faculty of Medicine, Ain Shams University, Cairo, Egypt; 2 Department of Psychiatry, Faculty of Medicine, Ain Shams University, Cairo, Egypt; 3 Luxor International Hospital, Luxor, Egypt

**Keywords:** Children with type 1-diabetes, Confidence in diabetes self-management, Health related quality of life, Continuous subcutaneous insulin infusion, Multiple daily injections

## Abstract

**Background:**

Novel innovations continue to emerge in type-1 diabetes (T1D) management aiming to improve glycemic control. Assessing the psychosocial outcomes of different treatment modalities is specifically crucial among children with T1D and differs from one population to another.

**Objectives:**

To compare the health related quality of life (HRQoL) and confidence in diabetes self-management (CIDS) among children with T1D on continuous subcutaneous insulin infusion (CSII) versus multiple daily injections (MDI) and to correlate them with the efficacy of glycemic control, Mini-International Neuropsychiatric Interview for Children and Adolescents(MINI-KID) depression module and socioeconomic-standard scale.

**Methods:**

This real life study (ClinicalTrials.gov number NCT04756011) included 60 children with T1D (30 on CSII and 30 on MDI), aged 6–18 years. Disease duration, insulin therapy, average self-monitoring of blood glucose (SMBG) and HbA1C were assessed. CIDS, socioeconomic-standard, MINI-KID depression and HRQoL scales were applied.

**Results:**

Children with T1D on CSII have significantly higher HRQoL and CIDS than those on MDI (P < 0.001). A significant negative correlation is found between HRQoL and insulin daily dose(P = 0.022), HbA1C(P < 0.001), average SMBG(P < 0.001) and MINI-KID depression scale(P < 0.001). A significant positive correlation is found between HRQoL and CIDS(P < 0.001) and health care, home sanitation, family possessions and occupation socioeconomic scores(P = 0.033, P = 0.001, P < 0.001 and P = 0.006, respectively). Multivariate regression analysis revealed that HRQoL is most associated with MINI-KID depression scale (P = 0.004) and annual total cost(P < 0.001).

**Conclusion:**

Children with T1D on CSII have significantly better HRQoL, CIDS and HbA1C with less depression than those on MDI.

## Highlights

- Children with T1D on CSII have significantly higher HRQoL and CIDS than those on MDI.
- HRQoL was positively correlated to CIDS and socioeconomic scales and negatively correlated to the MINI-KID depression scale, HbA1C and insulin daily dose.

## Introduction

Type 1 diabetes (T1D) is characterized by immune-mediated depletion of β-cells resulting in lifelong dependence on exogenous insulin [1]. The treatment of T1D and its complications impose a considerable burden on patients, health care providers and the society. Health related quality of life (HRQoL) combined with optimal glycemic control are recognized as integral targets in diabetes management [2].

Comparison of HRQoL in children with T1D and healthy peers has shown inconsistent results. Some studies reported lower HRQoL in children with T1D than their healthy peers [3]. Other studies found no difference between children with T1D and their healthy peers [4]. Studies suggest that male gender, longer diabetes duration, better glycemic control and higher socioeconomic status are associated with better HRQoL [5–7]. Less favorable HRQoL was associated with youths' perceptions that diabetes is upsetting, difficult to manage, and stressful, as well as fear of hypoglycemia [8, 9].

Currently, there is no cure for T1D; hence it is essential that insulin therapies are optimized to enable the best HRQoL for patients, while minimizing the risk of acute and long-term complications. The use of intensive insulin treatment regimens, in the form of multiple daily injections (MDI) and continuous subcutaneous insulin infusion (CSII; or insulin pumps), is associated with a reduction in the risk of developing long-term vascular complications in children with T1D [10].

However, data about the effect of CSII use on HRQoL are inconsistent. Non-randomized studies showed improvement in patients’ or parents’ ratings of HRQoL when patients were switched from MDI to CSII [11], but a study where 26 children were randomized to MDI or CSII for six months did not demonstrate a change in parents’ rating of HRQoL from baseline [12].

Effective diabetes management is a complex process requiring active behavioral involvement of children with T1D and their caregivers on a day-to-day basis. A key factor in achieving these behavioral goals is the individual’s confidence in his or her ability to perform specific tasks required to reach the desired goal [13]. The value of confidence in diabetes self-care (CIDS) in predicting self-care behaviors and outcomes in patients with diabetes is supported by several studies, in which CIDS was associated with better adherence, glycemic control, mental health, and social functioning [14]. However, no previous studies compared the CIDS between children with T1D on MDI and CSII.

Hence we aimed to compare the psychosocial impact (in terms of HRQoL and CIDS) of CSII versus MDI under real-life conditions in children with T1D in Egypt and to correlate it with the efficacy of glycemic control, depression and socioeconomic standard scale.

## Methodology

This case–control study was approved from the local ethical committee of Ain Shams University and registered in ClinicalTrials.gov (study number NCT04756011). Sixty children with T1D were recruited from the regular attendees of the Pediatric Diabetes Clinic, Pediatric Hospital, Ain Shams University. The study protocol was approved by the Ethical Committee of Ain Shams University, and an informed consent was obtained from each patient or their legal guardians before participation. Reporting of the study conforms to Consolidated Standards of Reporting Trials 2010 statement [15].

Patients were defined according to the criteria of the ISPAD 2018 [16]. Inclusion criteria were patients with T1D on insulin therapy, aged 6–18 years on regular insulin therapy with insulin pump or MDI for at least 1 year. Exclusion criteria included patients with other medical conditions (i.e. celiac disease or autoimmune thyroiditis), patients with other types of diabetes mellitus (i.e. maturity onset diabetes of youth (MODY) and type 2 diabetes mellitus) and patients with history of psychiatric disorders.

All included children were subjected to detailed medical history with special emphasis on age at onset of diabetes, disease duration, insulin therapy, history of acute complications i.e. frequency of hypoglycemia and number of hospital admission by diabetic ketoacidosis (DKA) and history of chronic micro and macro-vascular complications. Thorough clinical examination was done laying stress on anthropometric measures with calculation of standard deviation score and body mass index (BMI) measured as kg/m2 [17]. Peripheral blood samples were collected on potassium-ethylene diamine tetra-acetic acid (K2-EDTA) in sterile vacutainer tubes (final concentration of 1.5 mg/mL) (Beckton Dickinson, Franklin Lakes, NJ, USA) for assessment of HbA1C.

### Psychosocial assessment

The Diabetes self-management questionnaire was used to assess diabetes education. It is a validated 16 item questionnaire that assesses self-care activities associated with glycemic control. It includes four subscales; glucose management, dietary control, physical activity and health-care use; as well as a sum scale as a global measure of self-care [18].

The family socioeconomic level was assessed using the validated arabic socioeconomic level scale for health research in Egypt. It is a scale with 7 domains (education and culture, family, economic, occupation, family possessions, home sanitation and health care) with a total score of 84. The parental educational status was assessed as the educational level of both parents or caregivers [19].

The Confidence in diabetes self-care questionnaire (CIDS) scale was taken which included HbA1c, emotional distress, and fear of hypoglycemia, self-esteem, anxiety, depression, and self-care behavior. The CIDS scale is a reliable and valid measure of diabetes-specific self-efficacy for use in patients with type 1 diabetes. High psychometric similarity allows for cross-cultural comparisons [12].

Evaluation of health-related quality of life (HRQoL) was measured by the Peds-QL 4.0. The 23-item Peds-QL 4.0 generic core scales encompass (a) physical functioning (eight items), (b) emotional functioning (five items), (c) social functioning (five items), and (d) school functioning (five items). The Peds-QL 4.0 generic core scales comprised of parallel child self-report and parent proxy-report formats. This scale consistently facilitates the evaluation of the differences in HRQoL across young age groups, as well as tracking of HRQoL longitudinally. Scale scores are computed as the sum of the items divided by the number of items answered. The validated Arabic version was used with Cronbach’s α of the child and parent reports were greater than 0.70, for both instruments [20].

A validated Arabic version [21] of the Mini International Neuropsychiatric Interview for Children and Adolescents (MINI-KID), depression module [22] was used. The MINI-KID provides a structured interview for DSM IV and ICD-10 childhood and adolescent disorders that could be administered relatively quickly (∼ 25 min). It is suitable for administration to children from the age of 6 years to adolescence (up to 17 years, 11 months). Mood and suicidality modules were used.

### Annual cost

Data collection: Actual cost for insulin, CSII disposables and other drugs were collected from the hospital registry to avoid recall bias since, official sources of unit cost data for products in Egypt were not available. The cost of insulin was calculated as cost per average daily dose according to the patient′s information registered in his file in the past three months and extrapolated to 12 months. The annual cost of the CSII consumables was calculated according to medical records and the regimen followed by the patient. Data covered the 12-month period prior to the date of collection. The cost of healthcare resources (other than medication) used by patients was calculated by multiplying resource quantities by the average of available unit costs from the hospital accounting data. Hospitalization costs were obtained from the hospital data sources. These prices reflect real-world settings in Egypt. The base year for all costs was 2020. Bottom-up costing approach was used to estimate average annual costs per patient [23].

### Statistical analysis

Analysis of data was performed using software MedCalc v. 19. Quantitative variables were described as mean, standard deviation (SD), minimum and maximum. Qualitative variables were described as numbers (No.) and percents (%). Data were explored for normality using Kolmogorov–Smirnov test of normality. The results of Kolmogorov–Smirnov test indicated that most of data were normally distributed (parametric data) so parametric tests were used for most of the comparisons. Comparison between quantitative variables was carried out by One-way analysis of variance (ANOVA), which was used to test the difference between the means of several subgroups of a variable by pairwise comparisons. Comparison between qualitative variables was carried out by Chi-square test, which was used to test the statistical significance of differences in a classification system (one-way classification) or the relationship between two classification systems (two-way classification). Binary correlation was carried out by Pearson correlation test. Results were expressed in the form of correlation coefficient (R) and P-values. Multivariate linear regression analysis was used to assess factors independently associated with the HRQoL among the studied children with T1D on CSII. The confidence interval was set to 95% and the margin of error accepted was set to 5%, so the p-value was considered significant at a level of < 0.05.

## Results

Sixty children with T1D were enrolled; they were 32 males (53.3%) and 28 females (46.7%) with mean age 10.65 ± 3.17 years (range, 6–18). Their mean diabetes duration was 4.88 ± 2.05 years, range 1–11. Their daily insulin requirements ranged from 0.50 to 2.00 U/kg/day, with a mean ± SD of 0.97 ± 0.33. They were assigned into two groups; one group included children on CSII and the other group included children on MDI. Both groups were on their treatment regimen for at least 1 year.

### Psychosocial characteristics of the studied children with T1D

**Fig. 1:**
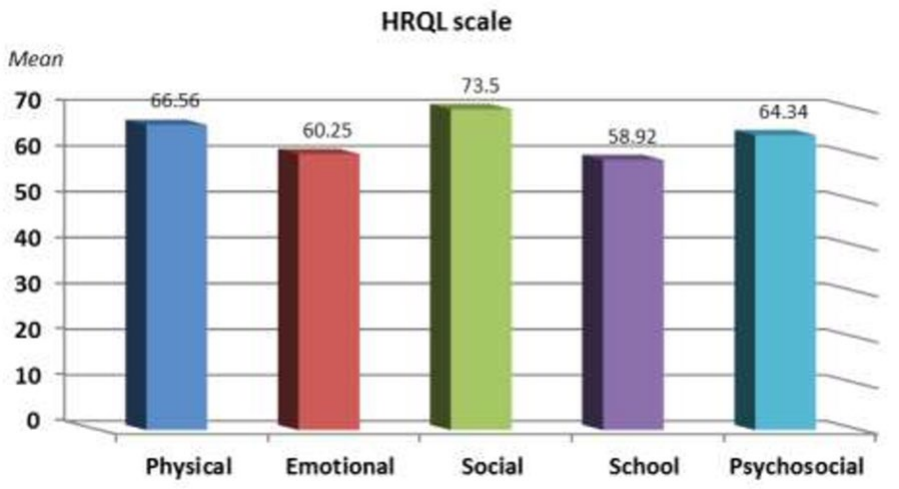
Health-related quality of life (HRQoL) of the studied children with type 1 diabetes (T1D)

**Fig. 2:**
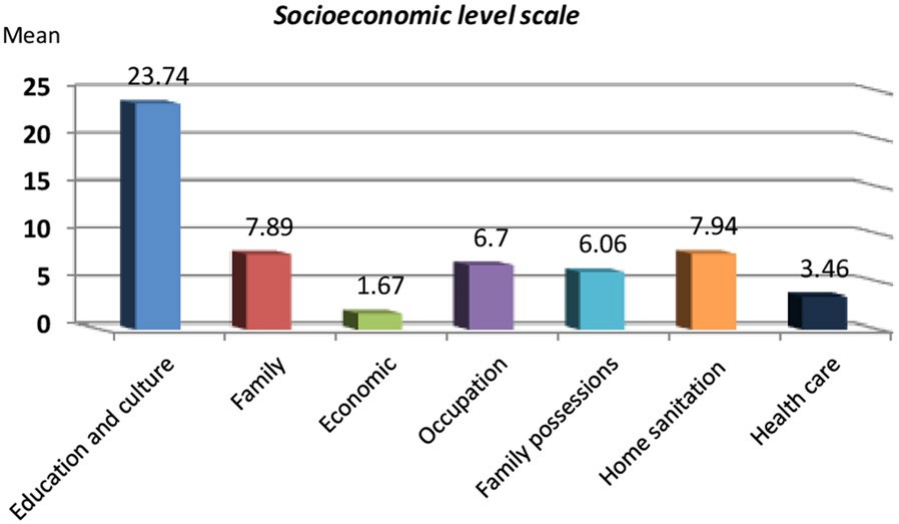
Socio-economic scale of the studied children with type 1 diabetes (T1D)

The mean CIDS scale of the studied children with T1D (n = 60) was 73.03 ± 9.84, range 51–89 and their mean total HRQoL was 64.71 ± 14.88, range 29.78–91.36, Fig. 1. Twenty two children had major depressive disorders (36.7%). Regarding socio-economic level scale, the mean education scale of the studied cohort was 23.74 ± 2.02, 16–28, their mean economic scale was 1.67 ± 0.80, 1–4, their mean family possessions scale was 6.06 ± 0.65, 5–7 and their mean health care score was 3.46 ± 0.92, 2–5, Fig. 2.

### Psychosocial characteristics and gender

Comparison of the psychosocial characteristics according to gender revealed no statistically significant difference in the socio-ecnomic level, CIDS and HRQoL among the studied males and females except for economic level which was higher among the studied females (P = 0.039).

### Psychosocial characteristics and CSII

Upon comparing children with T1D on CSII and MDI, children on CSII had significantly lower average total daily insulin dose (P = 0.007), SMBG readings (P < 0.001) and HbA1C (P < 0.001) than the MDI group, Table 1.

**Table 1:** Comparing the clinico-laboratory, psychosocial and cost data between the studied children with T1D on MDI and CSII

Clinical data	MDI (N = 30)		CSII (N = 30)		P value
	Mean	(SD)	Mean	(SD)	
Age (years) ^a^	10.70	3.42	10.60	2.95	0.904
Disease duration (years) ^a^	4.63	1.95	5.13	2.14	0.349
Insulin daily dose (u/kg/d) ^a^	1.09	0.33	0.86	0.29	**0.007**
Weight (Z score) ^a^	− 0.44	1.18	0.01	1.09	0.133
Height (Z score) ^a^	− 1.52	1.36	− 1.84	7.17	0.808
BMI (Z score) ^a^	0.51	1.20	0.48	0.75	0.921
HbA1C (%)^a^	8.87	1.02	6.94	1.09	** < 0.001**
SMBG (mg/dl) ^a^	187.50	63.44	107.53	33.26	** < 0.001**
Diabetes self-management questionnaire	4.10	0.06	5.30	0.05	** < 0.001**
Socioeconomic level scale^a^					
Education and culture	23.75	1.98	23.73	2.08	0.976
Family	8.00	0.66	7.80	0.89	0.362
Economic	1.21	0.51	2.03	0.81	** < 0.001**
Occupation	7.08	1.59	6.40	2.19	0.206
Family possessions	5.71	0.55	6.33	0.61	** < 0.001**
Home sanitation	7.38	1.21	8.40	1.19	**0.003**
Health care	3.79	0.72	3.20	1.00	**0.018**
Major depressive disorders (MINI-KID)^b^					
Positive (n %)	17 (56.7%)		5 (16.7%)		
Negative (n %)	13 (43.3%)		25 (83.3%)		**0.001**
HRQoL scale^a^					
Physical health	53.46	17.43	79.66	11.16	** < 0.001**
Emotional functioning	49.83	10.21	70.67	20.96	** < 0.001**
Social functioning	63.33	15.05	83.67	12.10	** < 0.001**
School functioning	47.17	8.68	70.67	13.63	** < 0.001**
Psychosocial health	53.69	6.91	74.99	12.40	** < 0.001**
CIDS scale^a^	66.73	7.08	79.33	8.06	** < 0.001**
Mean annual cost^a^	9536.28	3374.52	25,566.10	1401.92	** < 0.001**
Mean annual hospitalization cost^a^	1390.65	1610.41	330.33	820.89	**0.002**
Total cost^a^	9675.93	3465.86	25,599.43	1395.12	** < 0.001**

T1D: type 1 diabetes; MDI: multiple daily injections; CSII: continuous subcutaneous insulin infusion; BMI: body mass index; HbA1C: fraction C of glycated hemoglobin; SMBG: self-monitoring of blood glucose; HRQoL: health related quality of life; CIDS: confidence in diabetes self-management

^a^ANOVA test

^b^Chi-square test

P < 0.05 significant; P < 0.01 highly significant

Regarding the socioeconomic scale, children on CSII had significantly higher economic, family possessions, home sanitation and health care scales than those on MDI (P < 0.001, P < 0.001, P = 0.003 and P = 0.018, respectively). Moreover, they had significantly higher overall HRQoL, CIDS and diabetes self-management questionnaire score than the MDI group (P < 0.001), Table 1.

A significant negative correlation was found between total HRQoL and total insulin daily dose (P = 0.022), HbA1C (P < 0.001) and average SMBG readings (P < 0.001) Table 2.

**Table 2:** Pearson correlation (R) between HRQoL scale and clinico-laboratory and psychosocial data among the studied children with type 1 diabetes (T1D) (n = 60)

	HRQoL scale					
	Physical	Emotional	Social	School	Psychosocial	Overall
Age (years)						
R	− 0.048	− 0.23	− 0.048	− 0.007	− 0.144	− 0.113
P value	0.716	0.078	0.718	0.955	0.272	0.388
Disease duration (years)						
R	− 0.052	− 0.11	− 0.042	0.13	− 0.03	− 0.029
P value	0.695	0.403	0.752	0.323	0.818	0.825
Insulin daily dose (u/kg/d)						
R	− 0.094	**− 0.4**	− 0.192	**− 0.26**	**− 0.33**	**− 0.295**
P value	0.474	**0.002**	0.142	**0.045**	**0.01**	**0.022**
Weight Z score						
R	0.151	0.148	0.206	0.242	0.213	0.221
P value	0.248	0.258	0.115	0.062	0.103	0.09
Height Z score						
R	0.216	**0.374**	0.254	0.116	**0.304**	**0.298**
P value	0.097	**0.003**	0.051	0.378	**0.018**	**0.021**
BMI Z score						
R	0.002	0.136	0.15	0.024	0.117	0.099
P value	0.988	0.302	0.253	0.855	0.372	0.454
HbA1C (%)						
R	**− 0.561**	**− 0.359**	**− 0.502**	**− 0.437**	**− 0.489**	**− 0.549**
P value	** < 0.001**	**0.005**	** < 0.001**	**0.001**	**0.001**	** < 0.001**
SMBG (mg/dl)						
R	**− 0.495**	**− 0.442**	**− 0.363**	**− 0.413**	**− 0.473**	**− 0.513**
P value	** < 0.001**	** < 0.001**	**0.004**	**0.001**	**0.001**	** < 0.001**
Diabetes self-management questionnaire						
R	**0.677**	**0.441**	**0.585**	**0.55**	**0.605**	**0.6677**
P value	** < 0.001**	**0.0005**	** < 0.001**	** < 0.001**	** < 0.001**	** < 0.001**
Socioeconomic level scale						
Education and cultural						
R	0.125	0.044	0.259	0.172	0.169	0.175
P value	0.367	0.754	0.059	0.214	0.221	0.204
Family						
R	− 0.024	− 0.161	− 0.1	− 0.191	− 0.177	− 0.15
P value	0.861	0.247	0.47	0.167	0.201	0.279
Economic						
R	**0.543**	0.103	**0.402**	0.219	**0.277**	**0.367**
P value	** < 0.001**	0.46	**0.003**	0.112	**0.043**	**0.006**
Occupation						
R	− 0.081	− 0.059	− 0.066	0.032	− 0.041	− 0.054
P value	0.559	0.676	0.634	0.818	0.771	0.697
Family possessions						
R	**0.313**	**0.337**	**0.307**	**0.581**	**0.48**	**0.467**
P value	**0.021**	**0.013**	**0.024**	** < 0.001**	** < 0.001**	** < 0.001**
Home sanitation						
R	**0.454**	**0.375**	**0.313**	**0.371**	**0.417**	**0.459**
P value	**0.001**	**0.005**	**0.021**	**0.006**	**0.002**	**0.001**
Health care						
R	**− 0.413**	− 0.121	− 0.256	− 0.205	− 0.228	**− 0.291**
P value	**0.002**	0.384	0.062	0.136	0.097	**0.033**
CIDS						
R	**0.423**	**0.379**	**0.488**	**0.348**	**0.509**	**0.498**
P value	**0.001**	**0.003**	** < 0.001**	**0.007**	** < 0.001**	** < 0.001**

T1D: type 1 diabetes; HRQoL: health related quality of life; BMI: body mass index; HbA1C: fraction C of glycated hemoglobin; SMBG: self-monitoring of blood glucose; CIDS: confidence in diabetes self-management

R: Pearson correlation coefficient, P < 0.05 significant; P < 0.01 highly significant

A significant positive correlation was found between the overall HRQoL and each of the CIDS and diabetes self-management questionnaire (P < 0.001); with a significant positive correlation with the health care, home sanitation, family possessions and economic scores (P = 0.0325, P = 0.0005, P = 0.0004 and P = 0.0063, respectively), Table 2.

Multivariate logistic regression analysis for factors affecting the HRQoL revealed that it was most associated with the MINI-KID depression scale (P = 0.004), the diabetes self-management questionnaire (P = 0.05) and the annual total cost (P < 0.001), Table 3.

**Table 3:** Multivariate regression analysis of factors affecting HRQoL in the studied children with T1D (n = 60)

Variable	Total HRQoL scale		
	Coefficient (r)	R partial	P value
Age (years)	− 0.8273	− 0.1792	0.213
Duration of disease (years)	− 0.8181	− 0.1135	0.433
HbA1C (%)	1.3653	0.1180	0.414
Insulin daily dose (U/kg/day)	− 1.1995	− 0.04335	0.765
SMBG (mg/dl)	0.003333	0.01507	0.917
CIDS scale	− 0.06404	− 0.05178	0.721
MINI KID depression scale	− 8.2917	− 0.3254	**0.021**
Annual total cost (L.E)	0.001028	0.5274	** < 0.001**
Diabetes self-management questionnaire	51.5415	0.2787	**0.050**
R^2^ adjusted	0.617		
P value	** < 0.001**		

P < 0.05 significant; P < 0.01 highly significant

### Annual cost

Upon comparing children with T1D on CSII and basal bolus regimen, children on CSII had significantly higher mean annual cost than those on MDI (P < 0.001). Moreover, they had significantly lower mean annual hospitalization cost (P = 0.002) than the MDI group, Table 1.

## Discussion

A substantial progress has occurred in diabetes technology to allow people with T1D to more effectively and/or more easily achieve adequate glycemic control while enhancing their HRQoL. Evidence has supported the integral role of psychosocial factors in the management of T1D in children and adolescents [24].

Despite the implementation of the commercial use of CSII for individuals with T1D since the 1980s and the presence of literature supporting the glycemic benefits of CSII in people with T1D [25], the proportion of people using this technology still varies significantly from one country to another. Moreover, it might vary inside the same country according to the socioeconomic class. In Egypt, CSII is still self-funded; which limits the scale of its use in the community to those with high socioeconomic standard. In the United States of America, the pediatric diabetes consortium study found that pump therapy is more common in those with private health insurance, non-Hispanic white race, annual family income over $100,000, and a parent with a college education [26]. Similarly, another study involving 515 children with T1D showed that socioeconomic factors, namely, income and parental education and increased frequency of SMBG were predictive of CSII use [27]. This goes in concordance with the current study in which children on CSII had significantly higher economic level, family possessions, home sanitation and health care access than those on MDI. This might be attributed to the non-coverage of pump therapy by the health care/insurance system which likely influences the low adoption rates of this technology.

Distinguishing the HRQoL and CIDS of CSII versus MDI and their relation to glycemic control could help guide clinical centers and decision makers to choose the best therapeutic options. In the present study, children with T1D on CSII had significantly higher HRQoL and CIDS scores than those on MDI. Moreover, they had significantly lower HbA1C and average SMBG readings. In line with these results, a study involving adults with T1D found that CSII use contributes to marked HRQoL improvement [28]. In addition, the Danish national study on HRQoL in children with T1D on CSII and MDI showed better HRQoL in children on long term CSII [29]. Moreover, a recent survey of one of CSII systems suggested improved HRQoL and perceived control over diabetes in adults using CSII. From the 1245 adults with T1D included in the study, 53.5% indicated positive changes in their overall well-being, 72.5% perceived control over diabetes and 50.6% perceived hypoglycemic safety on CSII [30]. Hence, more studies addressing the effect of CSII use on the HRQoL and CIDS among children with T1D are needed.

Although the available data regarding CSII on HRQoL and CIDS in children are limited, this study found that CSII use has positive impact on HRQoL and CIDS. This is supported by a multi-centric randomized controlled trial on children with T1D from Germany that revealed improved HRQoL in children with T1D transitioned to CSII [31]. Similarly, another multi-centric quantitative correlational study showed improved HRQoL in adolescents on CSII [32]. This beneficial role of CSII on HRQoL and CIDS could be attributed to its ability to help children with diabetes and their parents feel less burdened and/or constrained by the day-to-day demands of diabetes, their feeling less restricted by the daily demands of diabetes and their better confidence in their ability to address hypoglycemia. However, this could be also attributed to the higher socioeconomic scale and lower depression scale among those on CSII. Hence, further prospective randomized studies are needed to verify the causal relationship between these covariables.

The relation between CSII and depression in children with T1D remains unraveled. In the current study, children with T1D on CSII had significantly lower depression score than those on MDI. This goes in agreement with Munkácsi and colleagues 2018 who found that adolescents with T1D on CSII had lower depression level than those on MDI [33]. These results reinforce the positive role of CSII in this vulnerable population.

In real life, where people with T1D face challenges of daily diabetes management, SC insulin and CSII could differ in terms of effectiveness, ease of use, and short and long term outcomes. However, out-of-pocket costs of CSII remain a crucial deterrent for large numbers of patients [34]. Real-world data on health care and societal costs of CSII compared with MDI therapy in children are scarce. In the current study, the mean annual cost of CSII was significantly higher among those on CSII. However; the mean annual hospitalization cost was significantly lower among those on CSII. Hence, more research should assess the cost effectiveness of CSII among this vulnerable population.

In the present study, HRQoL in children with T1D was independently associated with depression, diabetes self-management questionnaire and total annual therapy cost. This goes in agreement with Shapira and coworkers who found that the presence of comorbid psychological disorders was associated with lower HRQoL among children and adolescents with T1D [35]. This could be attributed to the poorer self-care, higher HbA1c and more hospitalizations for acute complications like diabetic ketoacidosis (DKA) among those with comorbid psychological disorders [36]. Moreover, children with T1D on CSII having better diabetes education were found to have better glycemic control [37]. This highlights the importance of offering optimum psychotherapy, diabetes education and medical care to these children.

The main limitations of the current study are its cross-sectional nature which could not imply causality and its small sample size. Another limitation is that the patients had to pay for the CSII as this treatment is not reimbursed in Egypt; therefore the HRQoL was affected by the socioeconomic scale of the patients and their families. Hence, further larger randomized prospective studies are needed to identify the HRQoL, CIDS and cost effectiveness of CSII versus MDI among children with T1D.

## Conclusion

Children with T1D on CSII have significantly better HRQoL, CIDS, depression scale and HbA1C with better economic and family possessions, home sanitation and access to health care facilities. Although mean annual cost was higher in children with T1D on CSII, they had significantly lower mean annual hospitalization cost.

## Data Availability

Data will be available from the authors upon request.
